# Disaster preparation in kidney transplant recipients: a questionnaire-based cohort study from a large United States transplant center

**DOI:** 10.5414/CN109280

**Published:** 2017-10-26

**Authors:** Shimi Sharief, Daniel Freitas, Deborah Adey, James Wiley

**Affiliations:** 1 Sutter West Bay Medical Group,; 2 UCSF Department of Family and Community Medicine,; 3 UCSF Division of Transplant Nephrology, and; 4 UCSF Institute for Health Policy Studies, San Francisco, CA, USA

**Keywords:** kidney transplantation, community surveys, disaster planning

## Abstract

Background: Few quantitative assessments have assessed disaster preparation in kidney transplant patients. This is a survey-based assessment of disaster preparedness of 200 patients at the University of California San Francisco, USA. Materials and methods: Patients answered questionnaires assessing their level of preparedness as well as barriers to preparation. Preparedness was scored based on response to 7 questions. Univariate analyses compared participant characteristics extracted from the medical chart against three tertiles of preparedness: low (scores 0 – 2), medium (scores 3 – 4), and high (scores 5 – 7). California counties were coded and mapped by average preparedness scores. Results: Only 30% of patients were highly prepared for disasters. Participants were prepared with available medication for 2 weeks (78.5%) and least prepared in having a medical ID bracelet (13%). Significant minorities of patients (40% of patients or more) were unprepared with lists of medications, important phone numbers and disaster kits. Preparedness was not associated with demographic and clinical characteristics. Monterey County was the most prepared of the 31 California counties sampled (score of 4.25 out of 7). Conclusion: All patients should be educated regarding disaster preparation. County and medical services should collaborate to address specialized populations in general preparedness planning.

## Introduction 

In the decade since Hurricane Katrina, due to the large numbers of dialysis-related morbidity and mortality in that event, there has been increasing attention on the topic of disaster preparation in those with kidney disease and other vulnerable populations [1]. National organizations such as Kidney Community Emergency Response (KCER) Coalition set-up in partnership with the Centers for Medicare and Medicaid Services (CMS) and other dialysis providers including Davita and Fresenius, have been actively involved with regulating and mandating evaluations and response capacity in dialysis providers in the setting of a disaster [2]. 

Another vulnerable population that has not received as much attention in the time since Katrina in 2005 are kidney transplant patients, who rely on specialized medication to prevent rejection and ensure the longevity of their grafts. Questionnaire-based studies have evaluated emergency preparedness in several populations: dialysis patients since Hurricane Sandy [3], type I diabetic children since Hurricanes Ike and Sandy in Texas [4], and the elderly in an assessment conducted in Akron, OH, USA [5]. Only one small study in Japan in the wake of the East Japan Quake has ever assessed disaster preparation in kidney transplant patients [6]. 

Unanswered questions remain including what is the state of preparedness in this population, who is prepared, their demographic characteristics and the communities they live in, and why they may be prepared (or not). In a single-center study conducted at the University of California at San Francisco (UCSF), we evaluated the emergency preparedness of a cohort of 200 participants recruited from the kidney transplant clinic to answer some of these questions. 

## Materials and methods 

The Committee on Human Research at UCSF approved the study under proposal number 16-019991. A survey instrument that arose from a prior qualitative analysis with a small group of 10 transplant patients was thoroughly pretested for clarity, word choice, topic flow and relevance in a small pilot sample of 18 participants and finalized and approved for the cohort of this study. Guidelines published by the National Kidney Foundation (NKF) and Centers for Medicare and Medicaid (CMS) informed the content of the questionnaire, in particular the sections within the NKF handbook devoted to transplant patients [7, 8]. 

The sample was a convenience sample of patients attending the transplant clinic for their follow-up appointments between September 8 and December 22, 2016, approached at random in the clinic waiting room prior to their appointments. Patients completed the self-administered survey while they waited for their appointments. Out of 844 unique patients present during the clinic visits for the duration of the study, 10 were ineligible to participate due to prior participation in either our qualitative or pilot work. 200 participants completed the survey. The consent process took ~ 10 – 15 minutes to complete and participants took between 5 and 10 minutes to complete the 16-question survey. 

Data was transcribed from the original paper and pen format to an online database on UCSF’s licensed version of REDCap (University of Vanderbilt, TN, USA). Two researchers entered the data which was then compared and validated for accuracy using the dual-data entry module on REDCap. Medical data was collected from the electronic health record by the primary researcher and entered directly into a REDCap data entry form. 

The questionnaire constitutes three major sections, the full text of which is available in Supplemental Material. A hypothetical scenario of a large earthquake that has hit the Bay Area frames the questionnaire and prefaces the questions to follow. Since the study population included those outside of high risk of earthquakes but still at risk for other natural disasters such as flash flood and wildfires, the scenario was clarified for the participant prior to the first question. The first section assessed confidence and medication-level preparedness. It also includes a question assessing perceived challenges during a disaster. The second section included questions intended to assess general preparedness including whether participants had a medical ID bracelet, a list of medications, and phone numbers to both their transplant physician and pharmacy as well as a disaster kit and a designated “meeting place” for evacuations as well as a question assessing what barriers might impede their personal disaster preparation. The third section asked specific questions regarding where they obtained information related to disasters, the presence of prior or simultaneous organ transplants, their diabetic status and if they carried enough of their diabetes medications in case of an emergency, as well as their educational level and age. Participants signed medical information release forms for access to their medical record permitting chart abstraction which provided the remaining medical and demographic details used in our study. 

Our primary hypothesis in recruitment was that time since transplant would be a significant predictor of preparedness with those patients who have had their transplants the longest being the most prepared. Power was calculated using G*Power 3.1 [9, 10]. Power for a test of the null hypothesis of equal mean preparedness in the three sample strata based on time since transplant ranges from 0.77 to 0.90 for n = 50 in each group, a probability of type I error = 0.05, and 1 – 2 point differences between the groups in number of preparedness items checked. We recruited a total of 200 patients based on this analysis. Statistical analyses were performed using STATA SE 14 (StataCorp, College Station, TX, USA) and results reported with chi square tests or Fisher’s exact tests, as appropriate. Figures were created using STATA and tables created using Excel 2013 (Microsoft, Redmond, WA, USA). Some analyses were verified in SPSS (IBM Corporation, Armonk, NY, USA). 

An α-level of 0.05 determined statistical significance. The analyses include cross-tabulations with χ^2^-statistics, t-tests, and analysis of variance. Geocoded maps were created in Google Fusion Tables (Google, CA, Mountain View, CA, USA) using ZIP code data from medical charts and coded to the respective county merged with publicly available county boundary map data. 

## Results 

Table 1 compares the distribution of patients based on major demographic characteristics in our sample to the overall clinic population of all surviving patients with a kidney transplant (Table 1). 

Of note, there were no differences in race/ethnicity distributions in our sample compared to the clinic population. Our sample was slightly younger than the clinic population with a larger proportion of patients under the age of 60. The sample and clinic population differ mostly in time since transplant. This is attributed to the fact that those longest from transplant see their transplant providers less frequently due to stability of allograft function over time. Additionally, the sample draws heavily from Medicare patients. This is explained by the fact that transplant patients are covered under Medicare for up to 3 years after their transplant after which this coverage lapses unless they qualify for reasons outside of their kidney transplant status (such as if they are over age 65 or have a permanent disability). The high prevalence of Medicare patients in our sample is therefore concordant with the relatively high percentage of those patients who are within 5 years of their transplant in our sample. 

**Figure 1.**
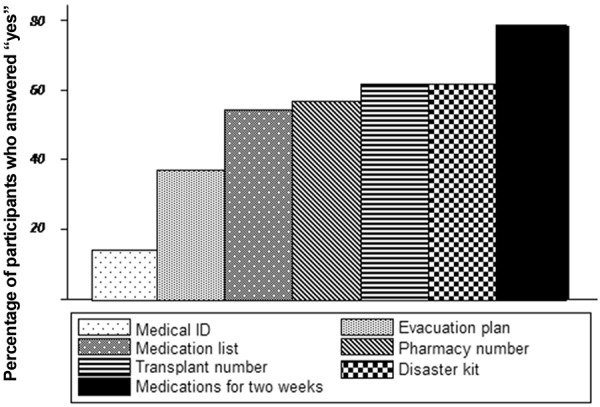
Bar graph of percentage of participants prepared for specific items on survey.

Participants answered objective assessments of their preparedness in the second section of the survey. Participants were most prepared in having 2 weeks of medication available as shown in Figure 1 and least prepared in having a medical ID bracelet that identifies them as transplant patients. A significant minority of patients (at least 40% of patients) were unprepared with lists of medications, important phone numbers, and disaster kits. 

**Figure 2.**
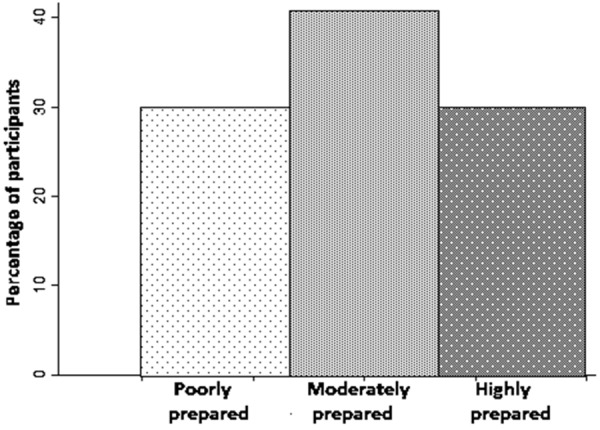
Histogram of participants in our sample based on preparedness level.

Using their responses from these survey items we created an index of preparedness which was then used to evaluate overall levels of preparedness in this population. The median preparedness score of participants was 4, with first and third quartiles at scores of 2 and 5, respectively. Dividing scores into tertiles of preparedness (low, medium, and high) shows that 29.5% of participants were poorly prepared (scores of 0 – 2), 40.5% were moderately prepared (scores of 3 – 4), and 30% were highly prepared (scores of 5 – 7) (Figure 2). 

Patients reported a variety of challenges in the setting of a disaster which thereby impacted their preparedness. The average preparedness scores were lower by 1 point in those who reported not knowing where to find help (2.7 vs. 3.7, p = 0.011) and not having enough medication (2.9 vs. 3.9, p < 0.001) or food (2.3 vs. 3.6, p = 0.011) as problems versus those who did not cite these reasons. Similarly, those who cited no challenges in the face of a disaster were almost 1 point higher than those who mentioned challenges to being prepared (4.1 vs. 3.1, p < 0.0001). 

Patients who reported certain barriers to preparation scored on average 1 whole point lower on preparedness than those who did not report barriers. This included those who reported not thinking about what they would need (2.6 vs. 3.6, p < 0.015), those who thought disasters were low probability (2.6 vs. 3.6, p = 0.015) and those who reported insurance barriers to filling medication (3.1 vs. 3.7, p = 0.047). 

In a univariate analysis between preparedness and various characteristics including demographic and clinical variables, none of the comparisons reached significance with p ≤ 0.05. There is a modest relationship between the ages of participants and their preparedness, with the participants in the highest preparedness category being slightly older than the participants in the other two groups (p = 0.11) (Table 2). Social demographics, such as marital and work status, were extracted from the chart and were not associated with preparedness. Similarly there was no association between having children and participant’s living situation and their preparedness (Table 2). 

There was a similar modest but not significant relationship between taking multiple medications and preparedness (p = 0.12) (data not shown). No associations were seen between multiple organ recipient status (such as liver, heart, or pancreas), rejection (either in the current or prior transplant), and preparedness and between preparedness and different types of immunosuppressive agents (steroid, calcineurin inhibitors, antimetabolites such as MMF and MPA, MTOR inhibitors, the injectable β-2 co-stimulation blockade agent belatacept). 

**Figure 3.**
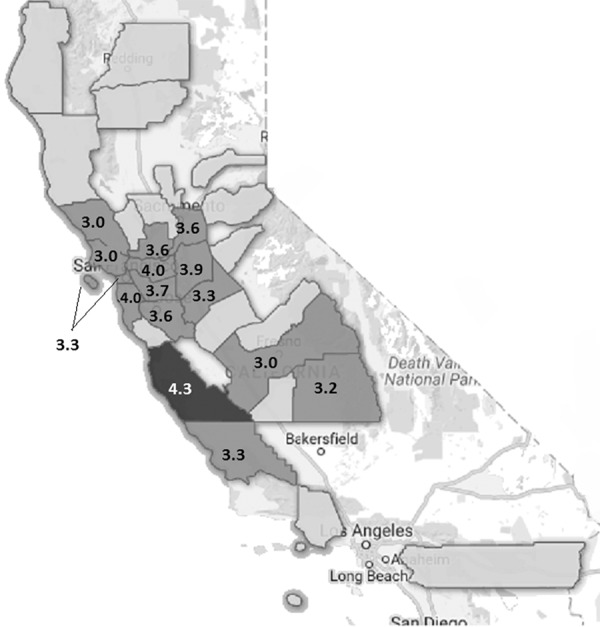
Map of all California counties assessed by average preparedness score.

Geocoded maps created with Google Fusion Tables compared counties based on preparedness. Participants in our study lived in 34 different counties, 2 of which were outside California, in Hawaii. Average scores for counties with at least 4 or more participants are indicated above the respective county on the map (Figure 3). An average score was not calculated for those counties represented by less than 4 participants. The three tertiles of preparedness were color-graded between low and high preparedness. The most prepared county with an average preparedness score of 4.25 was Monterey County, a coastal county in Northern California, while all the other counties were only moderately prepared with preparedness scores ranging between 2 and 4. 

All counties in the San Francisco Bay Area of Northern California were only moderately prepared for emergencies, the highest of which were Contra Costa and San Mateo counties’ average scores of 4.0 (with confidence interval (CI) 3.14, 4.78 and CI 3.17, 4.82 respectively), followed by Alameda at 3.7 (CI 3.13, 4.22) and Santa Clara County at 3.6 (CI 2.72, 4.48). San Francisco County had a mean preparedness score of 3.3 (CI 2.15, 4.34). 

## Discussion 

Based on our data, only 30% of all patients in the kidney transplant clinic were highly prepared for natural disasters. Those who were very highly prepared were not significantly different in demographics or clinical characteristics from those who were not highly prepared. This indicates that all patients regardless of gender, race, or other characteristics should be exposed to an educational curriculum consisting of information from the guidelines published by the National Kidney Foundation. This may be redundant information for only those 30% who are assessed to be highly prepared. 

It appears that there is a greater deficit for general preparedness information than for medical preparedness (having 2 weeks-worth of medication available) among transplant patients. This suggests that there is need for better education regarding general preparedness for an emergency or natural disaster such as that regarding the need for supplies of water, food, and establishment of a predesignated meeting place. From a medical standpoint, medical professionals may be unaware of the added risk of lack of preparation in this vulnerable population. Healthcare providers may be uneducated as to what information to provide to their patients to help them better prepare for an emergency. Those who reported feeling that disasters were low probability or have not thought about disaster preparation, despite living near active earthquake fault zones were less likely to be prepared than those who did not cite these two reasons. However, certain counties, such as Monterey County, appear to be better prepared than other surrounding counties: this could be due to its relatively high earthquake risk (99.4%), but it is no more earthquake-prone than many other counties in the San Francisco Bay Area (all greater than 90% with the exception of San Francisco county at 64%) [11]. This difference in emergency preparedness may be attributed to differences in educational outreach by local jurisdictions regarding emergency preparation. 

It is also important to note that while our study assessed how many patients have medication available on hand to last 2 weeks, it does not assess “active” stockpiling. Active stockpiling is the act of accumulating medications intentionally and storing them securely specifically in preparation for a disaster. The National Kidney Foundation recommendation on this topic is that patients store their medications in original bottles and if possible store 2 weeks extra of medications [8]. A large proportion of patients report barriers to filling prescriptions in a way that allows for husbanding an emergency supply, typically due to insurance issues. However, the majority of patients had 2 weeks of medication available which then raises some concern about non-adherence by the patient to the dose or frequency of medication. 

There were no demographic or clinical factors that were predictive for preparedness in the event of an emergency. Our hypothesis that time since transplant would influence preparedness did not hold true. It is also somewhat of a surprise that there seems to be no impact of number of medications, multiple organ recipient status, diabetic status, or age in being prepared for a natural disaster Earthquakes can have a devastating impact on a geographic region, but fortunately are rare events. The infrequency of natural disasters in northern California may influence the complacency of the population. Other natural disasters such as fire (forest fires) are fairly common in certain areas of Northern California, but may not be represented in this cohort. As people living in areas prone to forest fires and floods are more likely to have enough warning to be able to evacuate their home, one would think they might be better prepared to be able to leave quickly with an emergency bag. On the contrary, the infrequency of severe earthquakes combined with their unpredictability likely put disaster preparedness at lower priority. 

This is the first study of emergency preparedness conducted in this specialized patient population. Additional strengths include that despite prior survey-based assessments of preparedness in vulnerable populations such as in diabetic children as studied by Heptulla et al. [4] in Texas and Kadowaki et al. [6] in Japan, none of these studies have explored the reasons patients cite as barriers or challenges they face in being adequately prepared. Access to information and medication are unique barriers faced by those with chronic illness as evident in our data, and something that requires the combined efforts of both public health departments and medical personnel to address. 

This study is limited in its scope as a single-center study with a convenience sample of patients. While an in-person approach assisted in recruitment for the survey, this study fails to assess those patients who live further away and are not seen as frequently in the clinic. Financial constraints limited the ability to pay for interpretation services which would have aided in the recruitment of non-English speaking patients and assess their unique challenges faced in the setting of a disaster, such as for those in the Central Valley who primarily speak Spanish. There could be differences between those who did and did not complete the survey. Since we were not able to collect data on patients who refused participation, the social and clinical characteristics of these patients determining this selection bias are unknown. Future retrospective studies should target and assess these patients. 

A further limitation of our study is the inability of the question regarding medication availability to discriminate between those patients who passively vs. actively stockpiled medication. Additionally, it was apparent to the primary investigator during the final administration (after post-testing) that there was some variability in how patients were interpreting the wording of the question “normally on hand”. It appeared to the investigator in conversation with patients that some of them felt more confidently about their ability to possess 2 weeks of medication if they received 3-month refills. Those whose medication was filled on a monthly basis felt less comfortable with their ability given that 50% of the time they were less likely to have at least 2 weeks of medication unless they were diligent in requesting a refill early and if their insurance would approve that refills early. 

As evident in our sample and population demographics, transplant patients include groups of patients at high risk for adverse events during disasters: They are older, with multiple medical comorbidities and reliant on multiple medications. Elderly patients were the most vulnerable group in the evacuation efforts during Katrina, and most adults rely on at least one piece of durable medical equipment which makes them harder to evacuate [12]. Diabetic children have been shown to have adverse metabolic control in settings of disasters [13] and diabetic adults had worsening of their metabolic control after the 2011 Earthquake in Japan [14]. People with diabetes and heart failure were among those with the most problems for access to medication and care during Hurricane Katrina [15]. 

On a local level, our recommendation for transplant patients would be to prioritize self-preparation with medical ID bracelets, lists of medications as well as a supply of medication for 2 weeks. Additionally, because transplant patients rely on transplant providers (nephrologists and social workers) for a lot of their information – medical or not – providers should be provided basic emergency management skills to allow them to dispense some of this information. Office managers can sometimes be helpful liaisons to ensure institutional preparedness. Written material can also be helpful, and in our study population, we provided patients with a three-page guide for information in their area and a sample wallet card revised from the dialysis card published by KCER and available online on their website [16]. Additionally, we provided vendors for medical ID bracelets provided through a list on the National Kidney Foundation website who have low-cost programs [17]. Medical IDs and especially medical information located in one place was helpful and critical in timely care during Katrina. While we do not have a post-intervention assessment, the provision of information does address the primary barrier reported by patients that they do not know where to find relevant information. 

On a systematic level, several measures could help reduce the risk to vulnerable transplant patients in addition to their own efforts. In conversation with officials at KCER, we learned that during mass disasters, each ESRD Network is responsible for tracking dialysis patients that may be unaccounted for during an emergency or disaster and report any operational changes or patient access issues to KCER who then report to CMS. No such reporting requirement for transplant patients exists in a disaster-related capacity and similar processes could be life-saving in the setting of a future disaster. Medication management during large-scale disasters has been described as problematic [15] especially in those requiring diabetic agents [14]. Policy changes in determining insurance coverage for early refills are essential to supplementing these efforts. 

Future studies should focus on collaborative efforts between different California counties in addressing effective modes of communication and outreach to their respective constituents. The unavailability of a standard criterion or score defining “adequate preparation” is in itself an indication that this should be a focus of further research and inquiry. Future iterations of the questionnaire should also focus on assessing more social demographics within the questionnaire such as marital and work status as these might be incomplete in the medical record. Surveillance of preparation efforts should include ongoing evaluation of county level preparedness as well as ongoing outreach with updated information as they arise using the updated expert guidelines specific to a patient population as with National Kidney Foundation in transplant patients. 

## Acknowledgments 

We would like to acknowledge Claire Brindis DrPH, Director of the Phillip R. Lee Institute for Health Policy Studies for supporting this work with the help of the Jonathan Showstack Fund to cover the cost of recruitment materials including printed matter and gift cards for patient incentives. We would like to thank the staff at the Kidney Community Emergency Response Coalition for their input into survey design as well as important updates regarding ongoing kidney-related relief work being conducted nationally. We would also like to thank Dr. Naveena Bobba of the San Francisco Department of Public Health for her input regarding San Francisco wide measures and resources to provide our study patients throughout this project. 

## Funding 

This study was conducted while Dr. Sharief was a post-doctoral research fellow in the Division of Nephrology at UCSF. This work was supported by a grant from the National Institute of Diabetes and Digestive and Kidney Diseases (T32DK007219 supporting Dr. Sharief) and by Jonathan Showstack fund from the Institute for Health Policy Studies. The study funders had no role in study design, collection, analysis or interpretation of data, writing the report, or decision to submit the report for publication. 

## Conflict of interest 

All authors declare that they have no conflicts of interest. 

**Table 1.** Comparison of sample population to clinic population by major demographic variables.

Characteristic		Sample (%) (n = 200)	Population (%)	Population N (based on available data)
Age	≤ 60 years	127 (63.5)	4,003 (52.4)	7,632
	> 60 years	73 (36.5)	3,629 (47.6)	
Gender	Male	100 (50.0)	4,401 (57.7)	7,626
	Female	100 (50.0)	3,225 (42.3)	
Race	White	97 (48.5)	3038 (47.7)	6,376
	Black	32 (16.0)	780 (12.2)	
	Other	71 (35.5)	2558 (40.1)	
Primary insurance	Medicaid	16 (8.0)	238 (9.4)	2,525
	Medicare	82 (41.0)	483 (19.1)	
	Private	102 (51.0)	1,804 (71.5)	
Time since transplant	< 1 year	73 (36.5)	435 (5.0)	8,744
	1 – 5 years	77 (38.5)	1,437 (16.4)	
	> 5 years	50 (25.0)	6,872 (78.6)	

**Table 2.** Relationship between different demographic and clinical characteristics and level of preparedness.

		Preparedness categories			χ^2^/ Fisher	p-value
		Poor (n = 59)	Moderate (n = 81)	High (n = 60)		
Characteristic		Number of participants (%)				
Age	19 – 60	44 (34.65)	47 (37.01)	36 (28.35)	4.49	0.11
	> 60	15 (20.55)	34 (46.58)	24 (32.88)		
Race	White	32 (32.99)	38 (39.18)	27 (27.84)	2.94	0.57
	Black	8 (25)	11 (34.38)	13 (40.63)		
	Other	19 (26.76)	32 (45.07)	20 (28.17)		
Gender	Male	32 (32)	44 (44)	24 (24)	3.43	0.18
	Female	27 (27)	37 (37)	36 (36)		
Primary insurance	Medicaid	3 (18.75)	8 (50)	5 (31.25)	Fisher	0.91
	Medicare	25 (30.49)	33 (40.24)	24 (29.27)		
	Private insurance	31 (30.39)	40 (39.22)	31 (30.39)		
Education	Less than college	26 (28.89)	38 (42.22)	26 (28.89)	1.65	0.80
	College degree	22 (34.38)	23 (35.94)	19 (29.69)		
	Graduate/professional	11 (23.91)	20 (43.48)	15 (32.61)		
Time since transplant	Less than 1 year	23 (31.51)	25 (34.25)	25 (34.25)	2.10	0.72
	Between 1 and 5 years	21 (27.27)	34 (44.16)	22 (28.57)		
	Greater than 5 years	15 (30)	81 (40.5)	60 (30)		
Diabetes	Diabetic	15 (23.44)	26 (40.63)	23 (35.94)	2.28	0.32
	Nondiabetic	44 (32.35)	55 (40.63)	37 (27.21)		
Insulin preparation	Have insulin for 2 weeks	8 (18.18)	19 (43.18)	17 (38.64)	3.56	0.17
	Do not have for 2 weeks	2 (33.33)	4 (66.67)	0 (0.00)		
Marital status	Married	31 (26.27)	48 (40.68)	39 (33.05)	1.91	0.38
	Not married	28 (34.15)	33 (40.24)	21 (25.61)		
Work status	Working	22 (28.95)	31 (40.79)	23 (30.26)	Fisher	0.89
	Not working	37 (30.08)	50 (40.65)	36 (29.27)		
	Unknown	0 (0)	0 (0)	1 (100)		
Children	Have children	30 (27.52)	43 (39.45)	36 (33.03)	1.75	0.78
	No children	23 (32.86)	30 (42.86)	17 (24.29)		
	Unknown	6 (28.57)	8 (38.10)	7 (33.33)		
Living situation	Lives alone	9 (47.37)	4 (21.05)	6 (31.58)	4.53	0.34
	Lives with others	45 (28.13)	67 (41.88)	48 (30)		
	Unknown	5 (23.81)	10 (47.62)	6 (28.57)		

## Supplemental material

Supplemental material Study questionnaire
