# Supplementary material for: Disaster preparation in kidney transplant recipients: a questionnaire-based cohort study from a large United States transplant center
Source: Clin Nephrol. 2017 Oct 26;89(4):241–51. doi: 10.5414/CN109280 (PMC6102562; doi:10.5414/CN109280)
Supplement: Supplemental material [file clinnephrol-89-241-S01.pdf]

Supplemental material

STUDY QUESTIONNAIRE:

Subject #: ____________________

Imagine that a major earthquake has hit the Bay Area and you have survived without injury.
***For at least one week*, all public transportation is stopped, many roadways and bridges are blocked, and all cell phones and landlines are cut off. Most businesses, like grocery stores, banks and pharmacies, are closed.**
If there are no earthquakes where you live, imagine a flood, fire or other natural disaster.

1. How prepared are you to deal with **your needs as a transplant patient** after such a disaster? ***[Select the answer that BEST describes how you feel]***

□	a. I feel very confident that I could deal with ***all*** my needs as a transplant patient after a major disaster.
□	b. I could meet ***some but not all*** of my needs as a transplant patient after a major disaster.
□	c. I'm ***not*** really well prepared to take care of my needs as a transplant patient after a major disaster.

2. If it's difficult to fill prescriptions for transplant medications after a disaster; is the supply of medications that you ***normally*** have on hand enough to last for **two weeks**? ***[Select the answer that BEST fits your situation]***

□	**a. Yes,** I would have enough transplant medication to last for two weeks.
□	**b. No,** I would probably have to find an emergency supply of medication before the two weeks passed.

3. Which of these would be the biggest problems or challenges to you after such a disaster? ***[Select ALL that apply]***

□	a. I cannot walk long distances / I cannot walk at all
□	b. I will not know where to go to find help
□	c. I won't have enough medication/ I won't know how to replace lost or damaged medication
□	d. I won't have any food to survive
□	e. I depend on my family member to give me my medicine
□	f. Other problem (not listed above) **: Please specify:**
□	g. None of the above. I won't have any problems.

Now I would like to ask you some questions about information and resources that might help during an emergency like a major earthquake or other natural disaster.

4. Do you ***carry with you*** (not just by memory) a list that gives all your transplant medications by name and dose (e.g. on a card or paper where a rescue worker can find it)?

□ Yes	□ No

5. Do you ***wear*** a medical ID bracelet that identifies you as a transplant patient?

□ Yes	□ No

6. If phone lines were working, do you ***carry with you*** (not just by memory) the phone number of your pharmacy with you if you need it (e.g. on a card or paper where a rescue worker can find it)?

□ Yes	□ No

7. If phone lines were working, do you ***carry with you*** (not just by memory) the number of your transplant doctor with you in case you need it (e.g. on a card or paper where a rescue worker can find it)?

□ Yes	□ No

8. Do you have disaster kit at home in case of a major disaster? (For example, a disaster kit could include extra clothes and blankets, a supply of food and water, a battery-operated radio, flashlight and a first aid kit, ***together in one place***) ***[Select the BEST answer for you]***

□	a. Yes I have a disaster kit that includes ***ALL*** those items
□	b. I have a disaster kit with ***some but not all*** those items
□	c. I don't have a disaster kit with those items

↓

If you answered a or b, is your disaster kit "ready to go" in case you have to leave immediately (eg, in a backpack or other bag or container you can take with you quickly)?

□ Yes	□ No

9. Do you and your loved ones have a "meeting place" (a location to meet outside your home) in case of an emergency?

□ Yes	□ No

10. Do you believe you have all the resources you need in the case of a major disaster?

□	**a. Yes**, I have the resources I would need
□	**b. No**, I don't believe I have the necessary resources

↓

11. ***If you answered NO*** what are the most important reasons that you don't have all the resources you need after a major disaster? ***[Select ALL that apply]***

□	a. I have never thought about what I would need in a disaster
□	b. I think the chances of a major natural disaster are very small
□	c. I don't know where to get the information I might need
□	d. My insurance won't pay for extra medication /won't let me call in early for refills
□	e. Other: **Please specify:**

12. Where do you get information about your kidney transplant? ***[Select ALL that apply]***

□	a. Transplant doctor/nurse practitioner/pharmacist
□	b. General kidney doctor (nephrologist)/family doctor
□	c. Case worker/ social worker
□	d. Internet searches
□	e. National Kidney Foundation brochures
□	f. Patient support group
□	g. Friend(s) or family member(s)
□	h. Other: **Please specify:**

Now I would like to ask you a few questions about other medical conditions:

13. Do you have any other organ transplants? ***[Select ALL that apply]***

□	a. Heart transplant
□	b. Lung transplant
□	c. Liver transplant
□	d. Pancreas transplant
□	**e. Not applicable**

14. Are you diabetic?

□ Yes	□ No

↓

<u>IF YES:</u>

a. Do you use insulin?

□ Yes	□ No

b. Do you have two weeks supply of insulin ***<u>at all times</u>***?

□ Yes	□ No

I will finish with a few questions about you.

15. What is the **highest level** of education you have completed? ***[Select ONE answer]***

□	a. Grade/elementary school
□	b. Some high school
□	c. High school diploma or GED
□	d. Some college
□	e. College degree
□	f. Graduate or professional degree

16. What is your age? ***[Fill in]:*** _______________________
